# A tensor decomposition-based integrated analysis applicable to multiple gene expression profiles without sample matching

**DOI:** 10.1038/s41598-022-25524-4

**Published:** 2022-12-08

**Authors:** Y-h. Taguchi, Turki Turki

**Affiliations:** 1 Department of Physics, Chuo University, Tokyo, 112-8551 Japan; 2 Department of Computer Science, King Abdulaziz University, Jeddah, 21589 Saudi Arabia

**Keywords:** Computational biology and bioinformatics, Gene regulatory networks

## Abstract

The integrated analysis of multiple gene expression profiles previously measured in distinct studies is problematic since missing both sample matches and common labels prevent their integration in fully data-driven, unsupervised training. In this study, we propose a strategy to enable the integration of multiple gene expression profiles among multiple independent studies with neither labeling nor sample matching using tensor decomposition unsupervised feature extraction. We apply this strategy to Alzheimer’s disease (AD)-related gene expression profiles that lack precise correspondence among samples, including AD single-cell RNA sequence (scRNA-seq) data. We were able to select biologically reasonable genes using the integrated analysis. Overall, integrated gene expression profiles can function analogously to prior- and/or transfer-learning strategies in other machine-learning applications. For scRNA-seq, the proposed approach significantly reduces the required computational memory.

## Introduction

The integrated analysis of gene expression profiles is difficult to accomplish^1–4^. Its primary purpose is to compensate for small sample sizes by integrating gene expression profiles measured from multiple studies because the process is generally not straightforward, while matching samples is rare. In this study, sample matching refers to correspondence between independent studies. For instant, the straightest sample matching is control and treatment taken from the same individuals, e.g., younger age and older age. Or more weakly, two individuals with the same properties other than treatment, e.g., have the same weight, age, or sex. Sharing labeling is not sample matching, yet similar. For example, when healthy controls and disease patients are collected from two different hospitals, it is simple label sharing rather than matching. In this study, we considered the cases where even label sharing is missing; in this sense, removing batch effect is out of scope in this study. Batch effect refers to removing bias between two set of samples with the same labeling. For example, in two hospitals, A and B, healthy controls and disease patients should be collected. The difference between the two hospitals can be larger than that between healthy controls and patients in individual hospitals. This is referred to as “batch effect”, which must be removed before investigating the difference between patients and healthy controls common in two hospitals. Since we do not consider the cases that share labels, the batch effect is therefore not considered in this study. If sample-matching information is missing, and samples are not associated with common labeling (e.g., healthy controls or patients), the requirements of this process are not always fulfilled, even if we simply group gene expression profiles using their labels within individual sets. Hence, the establishment of general frameworks for integrating multiple gene expression profiles without sample matching and common sample labeling would be of considerable benefit^5^. Owing to these basic requirements, such methods must be unsupervised, since the integration of multiple gene expression profiles that share nothing other than the genes is impossible. In this study, we employ tensor decomposition (TD) for this purpose^6^. Integrating two independent studies without either sample matching or label sharing remains to be accomplished. Nevertheless, this kind of expectation often exist behind biological studies. For example, genes with different expression between healthy controls and patients can be compared with those whose expression vary with aging if the considered disease are related to aging. Directly integrating two gene expression profiles of comparison between healthy controls and patients and that of difference with aging would be promising, which is the primary aim of this study.

## Results

Figure S1 shows the workflow of analyses performed in this study.

### Integrated analysis of datasets 1, 2, and 3

First, to determine whether this simple idea can integrate multiple gene expression profiles lacking sample matching, we attempted to integrate datasets 1, 2, and 3. Datasets 1 and 3 comprised cell lines modeled as either healthy controls or ADs, whereas dataset 2 comprised cell lines aiming to mutate AD-associated genes indirectly related to AD-only. After obtaining a singular value vector, $u_{\ell _1 i}$, as described in Eq. (8), we first investigated whether they were associated with classifications. Singular value vectors attributed to the $j_k$th sample, $v_{\ell _1 j_k k}, 1 \le k \le 3$, were computed from $u_{\ell _1 i}$ using Eq. (10), and the coincidence of $v_{\ell _1 j_k k}$ with the classifications of datasets 1, 2, or 3 was investigated (Table S1 and Fig. S2).

$v_{\ell _1 j_k k}, 1\le \ell _1 \le 5$ were significantly correlated with classifications in any of datasets 1, 2, or 3. Next, we tried to determine $u_{\ell _1 i}$, which is used for gene selection, and we selected $u_{\ell _1 i}, 1 \le \ell _1 \le 5$ (see Table S5). $P_i$ are attributed to *i* using Eq. (9) while taking $\Omega _{\ell _1} = \{\ell _1 | 1 \le \ell _1 \le 5 \}$. The obtained $P_i$ were corrected using the BH criterion^6^, and 565 *i* (genes) associated with adjusted $P_i$ less than 0.01 were selected. To validate the selected 565 genes biologically, gene symbols were uploaded to Enrichr^7^. We observed various enrichments (Data S1). For example, the “KEGG 2021 Human” category listed six neurodegenerative disease-related pathways within the top-10 pathways (Table S2A). Similarly, the “Jensen Diseases” category listed four neurodegenerative diseases within the top-10(see Table S2A). Additionally, “Disease Perturbations from GEO down” and “Disease Perturbations from GEO up” listed similar neurodegenerative diseases within the top-ranked diseases (Data S1). We noted the “Allen Brain Atlas up” and “Allen Brain Atlas down” categories in which many AD-related brain regions were highly ranked (Data S1). Enrichment analyses using databases other than Enrichr also reported convincing results. The top- and the third-ranked terms in the “GAD_DISEASE_CLASS” category of DAVID^8,9^ were PSYCH and NEUROLOGICAL, respectively (Table S2A). The top-10 ranked terms in the “GAD_DISEASE” category of DAVID included five neurodegenerative diseases (Table S2A). g:Profiler^10^ listed 16 KEGG pathways as significantly enriched with gene symbols associated with 565 selected genes; 16 pathways included six neurodegenerative disease-related pathways (Table S2A). Thus, TD-based unsupervised feature extraction (FE) was used to integrate three gene expression profiles lacking sample matching, genes of expressions associated with classifications in three independent studies were identified, and genes selected were enriched by various neurodegenerative disease-related terms from three biological databases. This suggests that TD-based unsupervised FE is a promising method for integrating gene expression profiles without sample matching.

### Drug repositioning using the tensor obtained with datasets 1, 2, and 3

We have previously demonstrated^11^ that integrated analyses of gene expression profiles between model animals treated by various drugs and patients were useful to determining which drug compounds are effective against diseases. There, we integrated only one disease gene expression profile with that of the drug treatment. In the present study, we integrated more gene expression profiles of diseases with those of drug treatment because of the novel framework introduced herein. We employed the gene expression profile (i.e., the integration of datasets 1, 2, and 3, $x_{i\ell k}, 1 \le k \le 3$), and dataset 4 as a gene expression profile of drug treatment. We obtained $x_{i\ell 4}$, as described in Materials and Methods, to be integrated with $x_{i\ell k}, 1 \le k \le 3$, as obtained previously.  Higher-order singular value decomposition (HOSVD^6^) was applied to the obtained $x_{i\ell k}, 1 \le k \le 4$.

First, we sought to validate the singular value vectors, $v_{\ell _1 j_k k }$, associated with classifications (Table S1 and Fig. S3). It is obvious that they are coincident with the classifications of individual datasets. Then, we selected $u_{\ell _1 i}, 1 \le \ell _1 \le 5$ as those associated with $u_{\ell _2 \ell }, 1\le \ell _2 \le 4$ by investigating $G(\ell _1, \ell _2, \ell _3)$ in Eq. (8) while fixing $1 \le \ell _2 \le 4$ (see Table S6). $P_i$ are attributed to *i* using Eq. (9) while taking $\Omega _{\ell _1} = \{\ell _1 | 1 \le \ell _1 \le 5 \}$. The obtained $P_i$ were corrected using the BH criterion and 544 *i* genes associated with adjusted $P_i$ less than 0.01, as selected. To validate the selected 544 genes biologically, their associated gene symbols were uploaded to Enrichr. We then observed various enrichments (Data S2). For example, the “KEGG 2021 Human” category listed six neurodegenerative disease pathways within the top-10 pathways (Table S2B). Similarly, the “Jensen Diseases” category listed four neurodegenerative diseases within the top-10 diseases as well (Table S2B). In addition, “Disease Perturbations from GEO down” and “Disease Perturbations from GEO up” listed similar neurodegenerative diseases within the top-ranked diseases (Data S2). Upon examining the “Allen Brain Atlas up” and “Allen Brain Atlas down” categories, we observed that many AD-related brain regions were highly ranked (Data S2). Enrichment analyses using databases other than Enrichr also reported convincing results. The first- and fourth-ranked terms in the “GAD_DISEASE_CLASS” category of DAVID were PSYCH and NEUROLOGICAL, respectively (Table S2B). The top-10 ranked terms in the “GAD_DISEASE” category of DAVID included four neurodegenerative diseases (Table S2B). The g:Profiler listed 13 KEGG pathways as significantly enriched with gene symbols associated with 544 selected genes; 13 pathways included six neurodegenerative disease-related pathways (Table S2B). Hence, the TD-based unsupervised FE integrated four gene expression profiles lacking sample matching; it identified genes whose expressions were associated with classifications in three independent studies simultaneously; it selected genes that were enriched by various neurodegenerative disease-related terms from three biological databases.

Next, we attempted to identify how this strategy ranked drugs based upon gene expression profiles in dataset 4. After computing $v_{\ell _1 j^{[1]}_4 j^{[2]}_4 j^{[3]}_4 4} \in {\mathbb {R}}^{60617 \times 94 \times 4 \times 3}$ by Eq. (11), HOSVD was applied to $v_{\ell _1 j^{[1]}_4 j^{[2]}_4 j^{[3]}_4 4}$ with fixed $\ell _1$, and we obtained

1 $$\begin{aligned} v_{\ell _1 j^{[1]}_4 j^{[2]}_4 j^{[3]}_4 4} = \sum _{\tilde{\ell _1}=1}^{94} \sum _{\tilde{\ell _2}=1}^4\sum _{\tilde{\ell _3}=1}^3 {\tilde{G}}^{[\ell _1]} \left( {\tilde{\ell }}_1,{\tilde{\ell }}_2,{\tilde{\ell }}_3 \right) {\tilde{u}}^{[\ell _1]}_{{\tilde{\ell }}_1 j^{[1]}_4} {\tilde{u}}^{[\ell _1]}_{{\tilde{\ell }}_2 j^{[2]}_4} {\tilde{u}}^{[\ell _1]}_{{\tilde{\ell }}_3 j^{[3]}_4}, \end{aligned}$$

where ${\tilde{u}}^{[\ell _1]}_{{\tilde{\ell }}_1 j^{[1]}_4} \in {\mathbb {R}}^{94 \times 94}$ was a singular value vector attributed to drugs, ${\tilde{u}}^{[\ell _1]}_{{\tilde{\ell }}_2 j^{[2]}_4} \in {\mathbb {R}}^{4 \times 4}$ was a singular value vector attributed to dose density, and ${\tilde{u}}^{[\ell _1]}_{{\tilde{\ell }}_3 j^{[3]}_4} \in {\mathbb {R}}^{3 \times 3}$ was a singular value vector attributed to biological replicates. We checked the drugs having larger $\sum _{{\tilde{\ell }}_1=1}^5 \left( {\tilde{u}}^{[\ell _1]}_{{\tilde{\ell }}_1 j^{[1]}_4} \right)^2$, and Table S3 shows the top-five drugs for $1\le \ell _1 \le 4$. Because most were regarded as effective in the original study^12^, drug repositioning seems to have been successful.

### Transfer Learning  using the tensor obtained with datasets 1, 2, and 3

One possible application of the integrated analysis of multiple gene expressions lacking sample matching is analogous to transfer learning (TL)^13^, where pre-trained machine-learning models are used to achieve better performance, even with smaller sample sizes. Although deep learning (DL) is often used for TL, DL architectures are not suitable for gene expression profile processing. For example, Yifei et al.^14^ found that DL achieved better performance than linear regression (LR) only by less than 7 %. This can be attributed to the lack of structure for gene expression profiles. In contrast to images and sentences (or documents) to which DL was applied successfully, gene expressions are orderless vectors of real numbers. In contrast, TD was successfully applied to gene expression profiles^6^ because tensor similarly does not consider the order of real numbers at all. Thus, TD is a suitable architecture for processing gene expression profiles.

To determine whether the integrated analysis of multiple gene expression profiles obtained using datasets 1, 2, and 3 can be used as a pre-trained system in TL, we combined dataset 5 with $x_{i\ell k}, 1 \le k \le 3$ as a pre-trained system. Dataset 5 comprises gene expression profiles created by the overexpression of the ABCC1 gene, which was recently recognized as an Alzheimer’s disease (AD) therapy target^15^. Usually, only gene expression profiles caused by overexpression of ABCC1 gene are analyzed, and genes whose expression are altered by ABCC1 over-expression are selected. Then, selected genes are compared to those whose expressions are known to have been altered by AD. Nevertheless, this procedure is somewhat indirect because no sample matching between gene expression profiles of AD and ABCC1 overexpression experiments exist. Our strategy is more suitable for comparison between gene expression profiles of AD and ABCC1 over-expressions since these expression profiles can be directly compared.

After obtaining the TD as in Eq. (8) for dataset 5, $v_{\ell _1 j^{[1]}_5 j^{[2]}_5 j^{[3]}_5 5}$ was computed by Eq. (11); the correlation between six categories in dataset 5 and $v_{\ell _1 j^{[1]}_5 j^{[2]}_5 j^{[3]}_5 5}$ was then computed (Table S1), and the results are shown in Fig. S4. Although we have considered scenarios up to $\ell _1=5$, $v_{\ell _1 j^{[1]}_5 j^{[2]}_5 j^{[3]}_5 5}$ were coincident with classification. Thus, as expected, the data show correlations between gene expression profiles of AD and ABCC1 overexpression through TD.

We selected $u_{\ell _1 i}, 1 \le \ell _1 \le 5$ by investigating $G(\ell _1, \ell _2, \ell _3)$ in Eq. (8) and fixing $\ell _2 \in \{1,3,4,5\}$ (see Table S7). $P_i$ were attributed to *i* using Eq. (9) while taking $\Omega _{\ell _1} = \{\ell _1 | 1 \le \ell _1 \le 5 \}$. The obtained $P_i$ were corrected with the BH criterion and 660 *i* (genes) associated with adjusted $P_i$ less than 0.01 that were selected. To validate the selected 660 genes biologically, we uploaded gene symbols associated with genes to Enrichr and observed various enrichments thereof (Data S3). For example, the “KEGG 2021 Human” category listed six neurodegenerative disease-related (Table S2C) pathways within the top-10. Similarly, the “Jensen Diseases” category listed four neurodegenerative diseases (Table S2C) within the top-10. “Disease Perturbations from GEO down” and “Disease Perturbations from GEO down” listed similar neurodegenerative diseases within the top-ranked diseases (Data S3). In “Allen Brain Atlas up” and “Allen Brain Atlas down” categories, many AD-related brain regions were highly ranked (Data S3). Enrichment analyses using databases other than Enrichr also reported convincing results. The top-three terms in the “GAD_DISEASE_CLASS” category of DAVID were PSYCH, NEUROLOGICAL, and AGING (Table S2C). The top-10 terms in the “GAD_DISEASE” category of DAVID included four neurodegenerative diseases (Table S2C). The g:Profiler listed 16 KEGG pathways as significantly enriched with gene symbols associated with 633 selected genes; 16 pathways included six neurodegenerative disease-related pathways (Table S2C). Thus, we successfully selected genes whose expressions were simultaneously altered by both AD and ABCC1 over-expressions and biological properties evaluated by enrichment analysis.

### Integrated analysis of single-cell RNA-sequence (scRNA-seq) data

In this subsection, we applied our strategy to the integrated analysis of the scRNA-seq dataset. Because individual experiments with scRNA-seq included $\sim 10^4$ cells, it was unclear how to integrate multiple scRNA-seq profiles. After obtaining the TD, as shown in Eq. (8), where *k* was replaced with an integer, $1 \le c \le 25$, representing the *c*th RNA-seq measurement described in “Integrated analysis of scRNA-seq data” section, we focused on $u_{\ell _3 c} \in {\mathbb {R}}^{25 \times 25}$. Variable *c* was divided into four groups, including either AD or healthy controls in either hippocampus or cortex brain regions. Next, we determined which $u_{\ell _3 c}$ was associated with these four classes (Fig. S5). We found that only $u_{6 c}$ was associated with these four classes; categorical regression was applied to $u_{\ell _3 c}$ for each $\ell _3$ separately, and *P* values were computed and corrected by BH criterion. Only $\ell _3=6$ was associated with adjusted *P* values less than 0.05. The results showed that $\sum _{\ell _2} G(6 \ell _2 6)^2$ was the largest among $\sum _{\ell _2} G(\ell _1 \ell _2 6)^2$, where a summation was taken over $\ell _2$ since there is no reason to select one specific $\ell _2$ (Table S8). $P_i$ were attributed to *i* using Eq. (9) with $\Omega _{\ell _1} = \{\ell _1|~ 6\}$. $P_i$ were corrected, and 177 *i* (genes) were associated with an adjusted $P_i$ of less than 0.01. Gene symbols associated with these 177 *i* were uploaded to Enrichr (Data S4). The results were very distinct from those in Table S2. No neurodegenerative disease terms were detected within the top-10 ranked terms in either “KEGG 2021 HUMAN” or “JENSEN DISEASES” categories. Instead, the top-10 ranked terms in the “Human Gene Atlas” category contained several brain tissues (Table S4). In contrast, the “Disease Perturbations from GEO down” and “Disease Perturbations from GEO down” categories included many diseases related to the brain (Table S4).

Although these are only few examples, notwithstanding the simplicity of our strategy, it successfully listed genes associated with tissue specificity as well as AD. Therefore, our proposed strategy can deal with scRNA-seq very easily, even without directly considering the massive number of cells in individual scRNA-seq measurements because we consider only the top-10 singular value vectors within individual scRNA-seq gene expression profiles, including up to $10^4$ cells.

## Discussion

### Advantages of the proposed implementation

It is evident that our implementation has at least two unique advantages: First, it enables the integration of multiple gene expression profiles without sample matching. Datasets 1, 2, and 3 had sample sizes of 9, 23, and 8, respectively. Replacing these numbers of samples with those of singular value vectors (i.e., sample size of eight), we obtained a single tensor, $x_{i\ell k}$, to which the HOSVD was easily applied. It is also possible to evaluate the consistency of singular value vectors, $v_{\ell _1 j_k k}$, attributed to samples in individual datasets by computing it from $u_{\ell _1 i}$ with Eq. (10) after applying HOSVD to $x_{i\ell k}$ with classifications of individual datasets. Because this strategy is applicable to the general number of samples in individual gene expression profiles, it is very useful. Its second advantage is a reduction in the computational memory required for analysis. When we applied this implementation to scRNA-seq data, we employed only the highest-ranked 10 singular value vectors computed by applying singular value decomposition (SVD) to individual scRNA-seq data, rather than considering as many as $\sim 10^4$ cells. This drastically reduced the required memory by a factor of 100. Nevertheless, we successfully selected genes associated with various significantly enriched biological terms related to tissue specificity as well as diseases, as expected. Because more cells are expected to be sequenced in individual experiments via scRNA-seq in the future, our strategy is recommended for a wide range of scRNA-seq analysis applications.

### Visualization of relation between samples without sample matching

Figures S2, S3, and S4 show that the proposed strategy can relate gene expression profiles lacking sample matching with one another. For example, $v_{1 j_k k}$ are always coincident with classifications regardless of *k* or datasets considered. Furthermore, they look very similar among distinct integration (i.e., among Figs. S2, S3, and S4). Hence, profiles seen in $v_{1 j_1 1}$ that represent distinctions between AD and control in dataset 1 correspond to those seen in $v_{1 j_2 2}$ that represent the distinction between WT and the other three treated cell lines in dataset 2. This is a reasonable coincidence because the distinction between WT and treated cell lines is supposed to correspond to that between control and AD. Thus, our strategy can successfully relate gene expression profiles without apparent sample matching. Meanwhile, if we consider $v_{1 j_3 3}$, the situation differs a bit. Although we expect that $v_{1 j_3 3}$ represent distinctions between control and two AD cell lines, they represent the distinction between AD1 and the other two cell lines: AD2 and control. This suggests the possibility that AD2 fails to represent some property of AD. Usually, this kind of investigation is impossible, because we cannot compare two gene expression profiles without sample matching. Nevertheless, our strategy enables us to figure out the discrepancy in dataset 3 if it is compared with datasets 1 and 2. Although this is just an example, detailed comparisons of $v_{\ell _1 j_k k}$ between distinct *k* (i.e. datasets) allows for comparison of gene expression profiles even without sample matching.

### Comparisons with previous works

To demonstrate the superior performance of our proposed strategy, we compared the performance to some conventional methods that integrate multiple matrices. Most methods have been designed for genomic science require sample matching. For example, all nine methods listed in reference^16^ require sample matching. intNMF^17^ is also limited to datasets attributed to the same individuals. In contrast to these studies, although a review^18^ comprehensively reported on integrated multi-view analyses, samples were implicitly assumed to be shared, and no integrated methods aimed to integrate multiple matrices or tensors that did not share samples; only features were included. Although MINT^19^ was proposed to integrate gene expression profiles sharing genes rather than samples, the method assumed common labeling among multiple studies in a supervised learning framework. Conversely, its unsupervised framework required the common quantitative variables associated with all samples included in the studies; thus, this method cannot be applied to the present task of investigating the correlations between latent variables and classifications not shared between individual studies, as shown in Table S2. We could not find any implementations designed to integrate multiple gene expression profiles formatted as matrices or tensors sharing only genes and not samples. Thus, we sought suitable methods outside the genomic field. First, we applied CMF^20^, implemented as a CMF package in R^21^, to the integration of datasets 1, 2, and 3. We computed four latent variables assuming a Poisson distribution for gene expression. None of the obtained latent variables were significantly correlated with classification in datasets 1, 2, and 3 (Table S9). Then, we tried GFA^22^, which was also implemented as a GFA package in R. Although we computed five latent variables, none were correlated with the classification in dataset 1, although some were correlated with the classifications in datasets 2 and 3 (Table S9). Hence, these two advanced methods, CMF and GFA, failed to identify latent variables correlated with classifications of all three datasets using integrated analysis. Finally, we tried simple concatenation. Ironically, SVD applied to a $60617 \times 40$ contracted matrix comprising $x_{i j_1} \in {\mathbb {R}}^{60617 \times 9}$, $x_{i j_2} \in {\mathbb {R}}^{60617 \times 23}$ and $x_{i j_3} \in {\mathbb {R}}^{60617 \times 8}$ provided the first singular value vector, which was correlated with all classifications of the three datasets. However, the *P* values for the correlation with classification of dataset 1 were only very slightly significant ($P=0.05$, Table S9). We then selected 147 genes using Eq. (9) by replacing $u_{\ell _1 i}$ with the first singular-value vectors correlated with all classifications of datasets 1, 2, and 3. To confirm the inferiority of simple concatenations toward TD-based unsupervised FE, although we evaluated their enrichment by uploading 147 genes to Enrichr, DAVID, and g:Profiler, their enrichments were clearly poorer than those of genes selected by TD-based unsupervised FE (Table S2D and Data S5). Thus, we could not identify other methods comparable with or superior to TD-based unsupervised FE. Considering that the two advanced methods, CMF and GFA, were inferior to the SVD applied to simple concatenated matrices, the methods developed for this purpose (i.e., integrated analysis of multiple matrices) seem to be inapplicable to the present purpose. Hence, more advanced methods must be developed to be suitable for the purpose of the present work (i.e., integrated analysis of gene expression profiles that lack common matching samples).

### Treatment of missing values

An additional advantage of the proposed strategy may be noted as a by-product. scRNA-seq data is known to include many missing values, and matrix representation was previously employed to resolve this problem^23^. In the proposed strategy, although the employment of SVD and HOSVD did not aim to fill missing values, it should naturally function as a mechanism to do so. This may be the reason the present strategy also works for scRNA-seq data as well with neither any specific additional modifications nor implementations toward the treatment of scRNA-seq data set. Notably our proposed strategy successfully provided a platform to integrate typical gene expression profile measurements with small samples using scRNA-seq, which includes massive numbers of single cells, since both can be represented as a tensor form, $x_{ijk} \in {\mathbb {R}}^{N \times L \times K}$, no matter how large the number of cells included in the scRNA-seq. Because we can process scRNA-seq with $L=10$ in this study, this suggests the possibility that scRNA-seq does not provide the expected large amount of information according to the number of single cells.

### Comparisons with the original individual studies

Although we assessed original studies to evaluate how coincident individual studies are with the present integrated one, it was not easy since how the original study treated samples differed from the present study. For dataset 1, since original study^24^ did not selected genes whose expression differs between NDC and others, we could not compare their results with ours. For dataset 2, since original study^25^ did not simultaneously compare four groups of genes, but compared them only pairwisely, we could not compare their results with ours. For dataset 3, since original study^26^ did not distinguish between AD1 and AD2, we could not compare their results with ours. For datasets 4 and 5, since original studies^12,27^ compared treated cell lines with controls, which we did not try, we could not compare their results with ours. For dataset 6, since original studies^28^ did not compare AD samples and controls directly, but in only tissue specific manner, we could not compare their results with ours. In general, since the individual studies have their own purposes and compared samples along these specific lines while our methods are fully data driven, how samples are compared with hardly matches between individual studies and the present integrated study.

### Classification

In general, classification performance is less likely to be good, since by considering all datasets together, specificity to individual datasets is expected to decrease. Table S10 shows the classification performance achieved by $v_{\ell _1 j_kk}$ for integrated analysis of datasets 1, 2 and 3 in Table S1. As expected, the performance was not good. Thus, integrated analysis proposed in this study less likely achieves good classification performance.

### Comparison with principal component analysis applied to individual data sets

In order to determine how better integrated analysis can select common genes than separated analysis, we separately applied principal component analysis (PCA) to datasets 1, 2 and 3 so that PC scores are attributed to individual genes. Then, 100 top ranked genes with larger absolute PC scores are selected; Fig. S6 shows the Venn diagram. Although the number of genes selected by TD and shared with PCA for the first, second, and third PCs is not small, there are very few genes shared with PCA for the fourth and the fifth PCs. Since the fourth and the fifth components are often coincident with classification for more than one dataset with high significance (see Table S1), they are very important: the integrated analysis clearly allows for the identification of more common genes for datasets 1, 2, and 3 than separated analysis. In addition to this, PC scores attributed to genes by PCA are neither well correlated with one another nor correlated with $u_{\ell _1i}$ computed by TD (Table S11). This suggests that not only top ranked genes but also over all gene expression profiles are hardly similar with one another if PCA is applied to individual datasets. Thus integrated analysis using TD that allows us to have unique $u_{\ell _1 i}$ valid for all three datasets is useful.

### Conclusion

In this study, we proposed a new strategy that integrates multiple gene expression profiles that lack both sample matching and common labeling. This strategy successfully integrated AD gene expression profiles that lacked sample matching and AD scRNA-seq datasets. The former can be used for drug repositioning and TL. Massive amounts of computational memory can be saved with the latter. The proposed strategy appears to be useful for integrating gene expression profiles, even those lacking both sample matching and common labeling among them.

## Methods

### Mathematical formulations

#### Integration of multiple gene expression profiles with TD

Here, we consider cases in which the number of genes, *N*, is much larger than the number of samples in the *k*th gene expression profile, $M_k$, as $N \gg M_k$. Given $K, \; (1 \le k \le K)$ gene expression profiles represented as a matrix,

2 $$\begin{aligned} x_{ij_k} \in {\mathbb {R}}^{N \times M_k}, \end{aligned}$$

or a $(S+1)$-mode tensor,

3 $$\begin{aligned} x_{ij^{[1]}_kj^{[2]}_k \cdots j^{[S]}_k} \in {\mathbb {R}}^{N \times M^{[1]}_k \times M^{[2]}_k \times \cdots \times M^{[S]}_k}, \end{aligned}$$

where $1 \le s \le S$ stands for the *s*th experimental condition in the *k*th gene expression profile. Then, SVD or HOSVD^6^ is applied to obtain

4 $$\begin{aligned} x_{ij_k} = \sum _\ell u^{[k]}_{\ell i}\lambda ^{[k]}_\ell v^{[k]}_{\ell j_k}, \end{aligned}$$

where $u^{[k]}_{\ell i} \in {\mathbb {R}}^{ M_k \times N}$ are eigenmatrices attributed to genes . $v^{[k]}_{\ell j_k} \in {\mathbb {R}}^{M_{ k} \times M_{ k}}$ are eigenmatrices attributed to samples, $\lambda ^{[k]}_\ell$ are eigenvalues. The reason why we did not write $v_{\ell j }$ but $v^{[k]}_{\ell j_k}$ is because $v_{\ell j}$ usually means that $v_{\ell j} \in {\mathbb {R}}^{L \times M}$, *i.e.*, *M* is independent of *k*, but in our case, $M=M_k$, which has *k* dependence. Therefore, $v_{\ell j }$ is not $v^{[k]}_{\ell j_k}$ since *j* is $j_k$ with *k* dependence. Alternatively,

5 $$\begin{aligned} x_{ij^{[1]}_kj^{[2]}_k \cdots j^{[S]}_k}= & {} \sum _{\ell _1 \ell _2 \cdots \ell _S \ell _{S+1}} G \left( \ell _1 \ell _2 \cdots \ell _S \ell _{S+1}\right) u^{[k]}_{\ell _1 j_k^{[1]}} u^{[k]}_{\ell _2 j_k^{[2]}} \cdots u^{[k]}_{\ell _S j_k^{[S]}} u^{[k]}_{\ell _{S+1 }i }, \end{aligned}$$

where $G\left( \ell _1 \ell _2 \cdots \ell _S \ell _{S+1}\right) \in {\mathbb {R}}^{M^{[1]}_k \times M^{[2]}_k \times \cdots \times M^{[S]}_k \times N}$ is a core tensor representing a weight of products, $u^{[k]}_{\ell _1 j_k^{[1]}}$ $u^{[k]}_{\ell _2 j_k^{[2]}}$ $\cdots$ $u^{[k]}_{\ell _S j_k^{[S]}} u^{[k]}_{\ell _{S+1 }i }$, $u^{[k]}_{\ell _s j_k^{[s]}} \in {\mathbb {R}}^{M^{[s]}_k \times M^{[s]}_k}$, and $u^{[k]}_{\ell _{S+1} i} \in {\mathbb {R}}^{N \times N}$ are singular value orthogonal matrices. Thus, we derive the reduced matrices as either

6 $$\begin{aligned} x_{i\ell k} = \sum _{j_k=1}^{M_k} x_{ij_k} v^{[k]}_{\ell j_k} , 1 \le \ell \le L, \end{aligned}$$

or

7 $$\begin{aligned} x_{i\ell k}= & {} \sum _{j^{[1]}_k=1}^{M^{[1]}_k} \cdots \sum _{j^{[S]}_k=1}^{M^{[S]}_k} x_{ij^{[1]}_kj^{[2]}_k \cdots j^{[S]}_k} u^{[k]}_{\ell _1 j_k^{[1]}} \cdots u^{[k]}_{\ell _S j_k^{[S]}}, 1 \le \ell _s \le L^{[s]}_k (\le M^{[s]}_k) , 1 \le \ell \le L= \prod _{s=1}^S L^{[s]}_k. \end{aligned}$$

This implementation involves a problem of note. For example, in Eq. (4), simultaneous changes, $u^{[k]}_{\ell i} \rightarrow -u^{[k]}_{\ell i}$ and $v^{[k]}_{\ell j_k} \rightarrow -v^{[k]}_{\ell j_k}$, also satisfy Eq. (4). Hence, we cannot fix the signs of $u^{[k]}_{\ell i}$ and $v^{[k]}_{\ell j_k}$. This does not present a problem if only individual gene expression profiles are investigated. However, if we need to compare multiple profiles, challenges might arise. For example, we compare multiple profiles in scRNA-seq data analysis in this study. Thus, we fix signs of $v^{[k]}_{\ell j_k}$ such that the correlation coefficients between $u^{[1]}_{\ell i}$ and $u^{[k]}_{\ell i}, k>1$ have positive values prior to the computations in Eq. (6).

HOSVD is applied to $x_{i\ell k} \in {\mathbb {R}}^{N \times L \times K}$ to obtain

8 $$\begin{aligned} x_{i\ell k} = \sum _{\ell _1=1}^N \sum _{\ell _2=1}^L \sum _{\ell _3=1}^K G(\ell _1 \ell _2 \ell _3) u_{\ell _1 i}u_{\ell _2 \ell } u_{\ell _3 k}, \end{aligned}$$

where $G(\ell _1 \ell _2 \ell _3) \in {\mathbb {R}}^{N \times L \times K}$ is a core tensor, and $u_{\ell _1 i} \in {\mathbb {R}}^{N \times N}$. $u_{\ell _2 \ell } \in {\mathbb {R}}^{L \times L}$ and $u_{\ell _3 k} \in {\mathbb {R}}^{K \times K}$ are singular value matrices that are orthogonal.

#### Gene selection

We can also select genes, *i*, by attributing *P* values to the *i*th genes while assuming $u_{\ell _1 i}$ obeys a Gaussian distribution,

9 $$\begin{aligned} P_i = P_{\chi ^2} \left[ > \sum _{\ell _1 \in \Omega _{\ell _1}} \left( \frac{u_{\ell _1 i}}{\sigma _{\ell _1}}\right) ^2 \right] , \end{aligned}$$

where $P_{\chi ^2}[>x]$ is the cumulative $\chi ^2$ distribution where the argument is larger than *x*, $\sigma _{\ell _1}$ is the standard deviation, and the summation of $\ell _1$ is taken over a set of $\ell _1$, $\Omega _{\ell _1}$, which is a set of $\ell _1$ selected as having larger $\sum _{\ell ,\ell _3} G(\ell _1 \ell \ell _3)^2$. $P_i$ are corrected via the Benjamini-Hochberg (BH) criterion^6^, and *i* is associated with adjusted *P* values less than 0.01.

#### Projection of individual gene expression profiles onto the space spanned by $u_{\ell _1 i}$

To determine how samples $j_k$ within each *K* gene expression profile are located in the space spanned by the obtained singular value vectors attributed to genes, $u_{\ell _1 i}$, we project individual gene expression profiles onto the space spanned by $u_{\ell _1 i}$ as

10 $$\begin{aligned} v_{\ell _1 j_kk} = \sum _{i=1}^N u_{\ell _1i} x_{ij_kk} \end{aligned}$$

or

11 $$\begin{aligned} v_{\ell _1j^{[1]}_kj^{[2]}_k \cdots j^{[S]}_k k} = \sum _{i=1}^N u_{\ell _1i} x_{ij^{[1]}_kj^{[2]}_k \cdots j^{[S]}_k}, \end{aligned}$$

where $v_{\ell _1 j_kk} \in {\mathbb {R}}^{N \times M_k \times K}$ and $v_{\ell _1j^{[1]}_kj^{[2]}_k \cdots j^{[S]}_k k} \in {\mathbb {R}}^{N \times M_1 \times \cdots \times M_S \times K}$ are regarded as coordinates of individual samples, $j_k$ or $j_k^{[s]}$, in the space spanned by $u_{\ell _1 i}$.

### Gene expression profiles

Herein, we specifically focused on AD. All gene expression profiles included in the six studies listed below are selected from the gene expression omnibus (GEO). Individual profiles are normalized to have zero mean and a standard deviation of one within individual profiles. Thus, when gene expression profiles are formatted as a matrix, $x_{ij_k}, \in {\mathbb {R}}^{N \times M_k}$ representing expression of the *i*th gene at the $j_k$th sample,

12 $$\begin{aligned} \sum _{i=1}^N x_{ij_k}= & {} 0, \end{aligned}$$

13 $$\begin{aligned} \sum _{i=1}^N x_{ij_k}^2= & {} N, \end{aligned}$$

and when gene expression profiles are formatted as a tensor, $x_{ij^{[1]}_k\cdots j^{[S]}_k} \in {\mathbb {R}}^{N \times M_k^{[1]} \times \cdots \times M_k^{[S]}}$, representing the expression of the *i*th gene at the sample annotated by indices $j_1, \ldots , j_S$, and

14 $$\begin{aligned} \sum _{i=1}^N x_{ij_k^{[1]}\cdots j_k^{[S]}}= & {} 0, \end{aligned}$$

15 $$\begin{aligned} \sum _{i=1}^N x_{ij_k^{[1]}\cdots j_k^{[S]}}^2= & {} N . \end{aligned}$$

#### Dataset 1: GSE160224

The dataset^24^ denoted as 1 (i.e., $k=1$) in this study comprises as few as nine samples, including nondemented controls (NDC), three APP duplications, and three isogenically corrected induced pluripotent stem-cell lines. In this study, three control samples are treated as controls, and the other six are treated samples when the coincidence to singular value vectors or latent variables are investigated, and genes whose expression are altered between these two classes are intended to be selected. Gene expression profiles are measured using RNA-seq technology, while genes are annotated using the Ensembl gene ID. The genes whose expressions are measured numbers as many as 58,302 in contrast to the small number of samples. As a result, gene expression profiles are formatted as a matrix, $x_{ij_1} \in {\mathbb {R}}^{58302 \times 9}$, representing expressions of the *i*th gene of the $j_1$th sample.

#### Dataset 2: GSE155567

The dataset^25^ denoted as 2 (i.e., $k=2$) in this study comprises four classes, including THP1 macrophages after the knockout of CD33 and/or the knockdown (silencing) of PTPN6 (six samples per class). Thus, in all, it includes as few as 24 samples. In this study, we do not specifically assume which classes are considered controls, but they are regarded as four categorical classes when the coincidence with singular value vectors or latent variables are investigated. For unknown reasons, although gene expression profiles of one sample are missing, it is unlikely to affect the outcome because it includes almost all (23 out of 24) samples. Gene expression profiles are measured using RNA-seq technology, and genes are annotated using the Ensembl gene ID. The number of genes whose expressions are measured amount to as many as 60,617 in contrast to the small number of samples. As a result, gene expression profiles are formatted as a matrix, $x_{ij_2} \in {\mathbb {R}}^{60617 \times 23}$, representing the expression of the *i*th gene of the $j_2$th sample.

#### Dataset 3: GSE162873

The dataset^26^ denoted as 3 (i.e., $k=3$) in this study comprises eight samples, four of which are AD cell lines, and the other four are normal. Because four AD cell lines comprise two sets of two samples using two distinct cell lines, this dataset includes three categorical classes when the coincidence with singular value vectors or latent variables is investigated. Two samples are taken from the first AD cell line, two samples are taken from the second AD cell line, and four samples are taken from normal cell lines. Gene expression profiles are measured by RNA-seq technology, and genes are annotated using the Ensembl gene ID. The number of genes whose expressions are measured amounts to as many as 47,749 in contrast to the small number of samples. As a result, gene expression profiles are formatted as a matrix, $x_{ij_3} \in {\mathbb {R}}^{ 47749 \times 8}$, representing the expression of the *i*th gene of the $j_3$th sample.

#### Dataset 4: GSE164788

The dataset^12^ denoted as 4 (i.e., $k=4$) in this study is an *in-vitro* differentiated mixture of neuron and glial cells derived from the ReNcell VM neural progenitor cell line treated with 80 different compounds; mRNA levels are measured using RNA-seq. Among the 80 compounds, we select 94 drugs and combinations from which at least two doses are tested. For each dose, three biological replicates are provided. When more than three are used, we randomly select three of them. Gene expression profiles are measured by RNA-seq technology, and genes are annotated using the Ensembl gene ID. The genes whose expressions numbers as many as 28,044. As a result, gene expression profiles are formatted as a tensor, $x_{ij^{[1]}_4j^{[2]}_4j^{[3]}_4} \in {\mathbb {R}}^{28044 \times 94 \times 4 \times 3}$, representing the expression of the *i*th gene at the $j_1$th drug combination, the $j_2$th dose, and the $j_3$th biological replicates.

#### Dataset 5: GSE164642

The dataset^27^ denoted as 5 (i.e., $k=5$) in this study comprises three sets of six samples; each includes two classes corresponding to ABCC1 activated cells by distinct RNA or control cells. They include three biological replicates (18 total samples). Gene expression profiles are measured using RNA-seq technology, and genes are annotated using the Ensembl gene ID. Thus, they are treated as six categorical classes when the coincidence with singular value vectors or latent variables is investigated. The genes whose expressions are measured numbers as many as 58,003 in contrast to the small number of samples. As a result, gene expression profiles are formatted as a tensor, $x_{ij^{[1]}_5j^{[2]}_5j^{[3]}_5} \in {\mathbb {R}}^{58003 \times 3 \times 2 \times 3}$, representing the expression of the *i*th gene at those treated by the $j^{[1]}_5$th RNA, the $j^{[2]}_5$th treatment ($j^{[2]}_5=1$:control, $j^{[2]}_5=2$:ABCC1 activated,) and the $j^{[3]}_5$th biological replicates.

#### Dataset 6: GSE163577

This is an scRNA-seq dataset^28^ denoted as 6 (i.e., $k=6$) in this study, including both AD and healthy controls from 25 hippocampus and superior frontal cortex samples across 17 control and eight AD patients. 25 individual datasets are formatted as a matrix, $x_{ij^{[c]}_6} \in {\mathbb {R}}^{33538 \times M^{[c]}_6}, 1 \le c \le 25$, representing the expression of the *i*th gene at the $j^{[c]}_6$th cell within the *c*th scRNA-seq profile. The number of cells, $M^{[c]}_6$, in individual datasets vary and is roughly $10^4$.

### Integrated analysis of gene expression profiles

#### Integrated analysis of datasets 1, 2, and 3

When we integrate the datasets 1, 2, and 3, we apply SVD to them to get

16 $$\begin{aligned} x_{ij_1}= & {} \sum _\ell u^{[1]}_{\ell i} \lambda ^{[1]}_\ell v^{[1]}_{\ell j_1} \end{aligned}$$

17 $$\begin{aligned} x_{ij_2}= & {} \sum _\ell u^{[2]}_{\ell i} \lambda ^{[2]}_\ell v^{[2]}_{\ell j_2} \end{aligned}$$

18 $$\begin{aligned} x_{ij_3}= & {} \sum _\ell u^{[3]}_{\ell i} \lambda ^{[3]}_\ell v^{[3]}_{\ell j_3} \end{aligned}$$

Then, we compute $x_{i \ell k}$ using Eq. (6) while setting $L=8$ because $M_1=9, M_2=24, M_3=8$, and *L* cannot exceed $M_k$. Because the number of genes whose expressions are measured differs among datasets 1, 2, and 3, we employ $N=60617$ as the number of genes whose expressions are measured in dataset 2 and the largest number of genes measured among datasets 1, 2, and 3. Then, these three gene expression profiles are formatted as a tensor,

19 $$\begin{aligned} x_{i \ell k} \in {\mathbb {R}}^{60617 \times 8 \times 3}, \end{aligned}$$

where the missing expressions in datasets 1 and 3 caused by the smaller number of genes than in dataset 2 are filled with zero. HOSVD is applied to $x_{i\ell k}$ as in Eq. (8).

#### Drug repositioning using the tensor obtained with datasets 1, 2, and 3

HOSVD is applied to $x_{ij_4^{[1]}j_4^{[2]}j_4^{[3]}} \in {\mathbb {R}}^{28044 \times 94 \times 4 \times 3}$. Then, $x_{i\ell 4}$ is computed using Eq. (7) with $L_4^{[1]} =4, L_4^{[2]}=2$, and $L_4^{[3]}=1$. The motivations of this choice are as follows. Biological replicates are expected to have common gene expression, and the first singular value vector, $u_{1 j^{[3]]}_4}$, is expected to have constant value regardless of $j^{[3]}_4$, based upon previous studies. Thus, it is sufficient to consider only $u_{1 j^{[3]}_4}$ for biological replicates. Then, two choices remain, including $L_4^{[1]} =4, L_4^{[2]}=2$ or $L_4^{[1]} =2, L_4^{[2]}=4$. Clearly, the former is reasonable because the number of drug combinations, at 94, is much larger than that of dose density, which is four. Missing values are filled with zero. In addition to $x_{i\ell k}, 1 \le k \le 3$, obtained in the previous subsection, HOSVD is applied to $x_{i \ell k} \in {\mathbb {R}}^{60617 \times 8 \times 4}$ as in Eq. (8).

#### TL using the tensor obtained with datasets 1, 2, and 3

To evaluate the performance of TL, HOSVD is applied to $x_{ij_5^{[1]}j_5^{[2]}j_5^{[3]}} \in {\mathbb {R}}^{58003 \times 3 \times 2 \times 3}$. Then, $x_{i\ell 5}$ is computed using Eq. (7) with $L_5^{[s]} =2, 1\le s \le 3$. HOSVD is applied to $x_{i \ell k} \in {\mathbb {R}}^{60617 \times 8 \times 4}$, obtained with Eq. (8) after $x_{i \ell 4}$ is replaced with $x_{i \ell 5}$.

#### Integrated analysis of scRNA-seq data

SVD is applied to $x_{ij^{[c]}_6}, 1 \le c \le 25$ one-by-one. Then, $x_{i \ell c}$ is computed by Eq. (6) while replacing *k* with *c* and $L=10$. Then, HOSVD is applied to $x_{i \ell c} \in {\mathbb {R}}^{33538 \times 10 \times 25}$.

### Methods to be compared

In the following, we tested three methods that assume the latent variables attributed to genes, *i*, which are also common among datasets 1, 2, and 3.

#### Collective matrix factorization (CMF)

To perform CMF, we used $x_{I j_1} \in {\mathbb {R}}^{60617 \times 9}$, $x_{i j_2} \in {\mathbb {R}}^{60617 \times 23}$, and $x_{i j_3} \in {\mathbb {R}}^{60617 \times 8}$, such that they shared the same number of genes. Missing values were filled with zero and were normalized to have zero mean and a standard deviation of one, as denoted in the beginning of this section. The structure of assumed modeling is given as

20 $$\begin{aligned} x_{i j_k} = \sum _{\ell =1}^L u_{\ell i} u^{[k]}_{\ell j_k} + b^{[k]}_i + b^{[k]}_{j_k} + \varepsilon ^{[k]}_{ij_k}, \end{aligned}$$

where $u_{\ell i} \in {\mathbb {R}}^{L \times 60617}$,$u^{[k]}_{\ell j_k} \in {\mathbb {R}}^{L \times M_k}$, and $b^{[k]}_i \in {\mathbb {R}}^{60617}$, $b^{[k]}_{j_k} \in {\mathbb {R}}^{M_k}$, $\varepsilon ^{[k]}_{ij_k} \in {\mathbb {R}}^{60617 \times M_K}$. We also employed an option wherein $x_{i j_k}$ obeys a Poisson distribution because the negative signed binary distribution usually assumed for the RNA-seq dataset is not provided as an option. Before applying this model to $x_{ij_k}$, some constants were added so that they do not take negative values, because Poisson distributions will not accept them. $L=4$ because TD-based unsupervised FE can identify a singular value vector correlated with classification in datasets 1, 2, and 3 within the top four.

#### Group factor analysis (GFA)

The datasets used were the same as those used in the trials using CMF. The structure of assumed model is given as

21 $$\begin{aligned} x_{i j_k} = \sum _{\ell =1}^L u_{\ell i} u^{[k]}_{\ell j_k} + \varepsilon ^{[k]}_{ij_k}. \end{aligned}$$

The primary difference from CMF, which employs Bayesian inferencing, is that GFA does not assume the distribution of $x_{i j_k}$. $L=5$ was assumed because it was employed in the example in the GFA tutorial and is larger than the number of singular value vectors computed with TD-based unsupervised FE correlated with classifications in datasets 1, 2, and 3.

#### Simple concatenation

A matrix, $x_{ij} \in {\mathbb {R}}^{60617 \times 40 }$, where $40 =\sum _{k=1}^3 M_K$, is generated by concatenating three matrices, $x_{ij_1}$, $x_{ij_2}$, and $x_{ij_3}$, to share the row number:

22 $$\begin{aligned} x_{ij} = \left\{ \begin{array}{clc} x_{i j_1}, &{} j_1=j, &{}1 \le j \le 9 \\ x_{i j_2}, &{}j_2=j-9, &{} 10 \le j \le 32 \\ x_{i j_3}, &{}j_3=j-32, &{} 33 \le j \le 40 \end{array} \right. \end{aligned}$$

Then, SVD was applied to $x_{ij}$ to obtain

23 $$\begin{aligned} x_{ij} = \sum _{\ell } u_{\ell i} \lambda _\ell v_{\ell j}. \end{aligned}$$

$v_{\ell j}$ for $1 \le j \le 9$ were used for the latent variables attributed to nine samples in dataset 1, $v_{\ell j}$ for $10 \le j \le 32$ were used for the latent variables attributed to 23 samples in dataset 2, and $v_{\ell j}$ for $33 \le j \le 40$ were used for the latent variables attributed to eight samples in dataset 3.

### Classification

Evaluation of classification performances were tested via linear discriminant analysis (LDA) using +lda+ function in MASS package in R^21^. Labels are treated as factors and prior probabilities of individual labels are set to be equal. Leave one out cross validation was employed with setting “CV=T” option.

### Source code

Sample R^21^ source code is available as supplementary material (R version 4.1.3).

## Supplementary Information

Supplementary Information 1.

Supplementary Information 2.

Supplementary Information 3.

Supplementary Information 4.

Supplementary Information 5.

Supplementary Information 6.

Supplementary Information 7.

Supplementary Information 8.

Supplementary Information 9.

## Data Availability

All data sets used in this study can be obtained via the NIH/NCBI Gene Expression Omnibus (GEO) repository using accession numbers GSE160224, GSE155567, GSE162873, GSE164788, GSE164642, and GSE163577.
