# Supplementary material for: A tensor decomposition-based integrated analysis applicable to multiple gene expression profiles without sample matching
Source: Sci Rep. 2022 Dec 8;12:21242. doi: 10.1038/s41598-022-25524-4 (PMC9732005; doi:10.1038/s41598-022-25524-4)
Supplement: Supplementary file 8 — Supplementary Information 8. [file 41598_2022_25524_MOESM8_ESM.pdf]

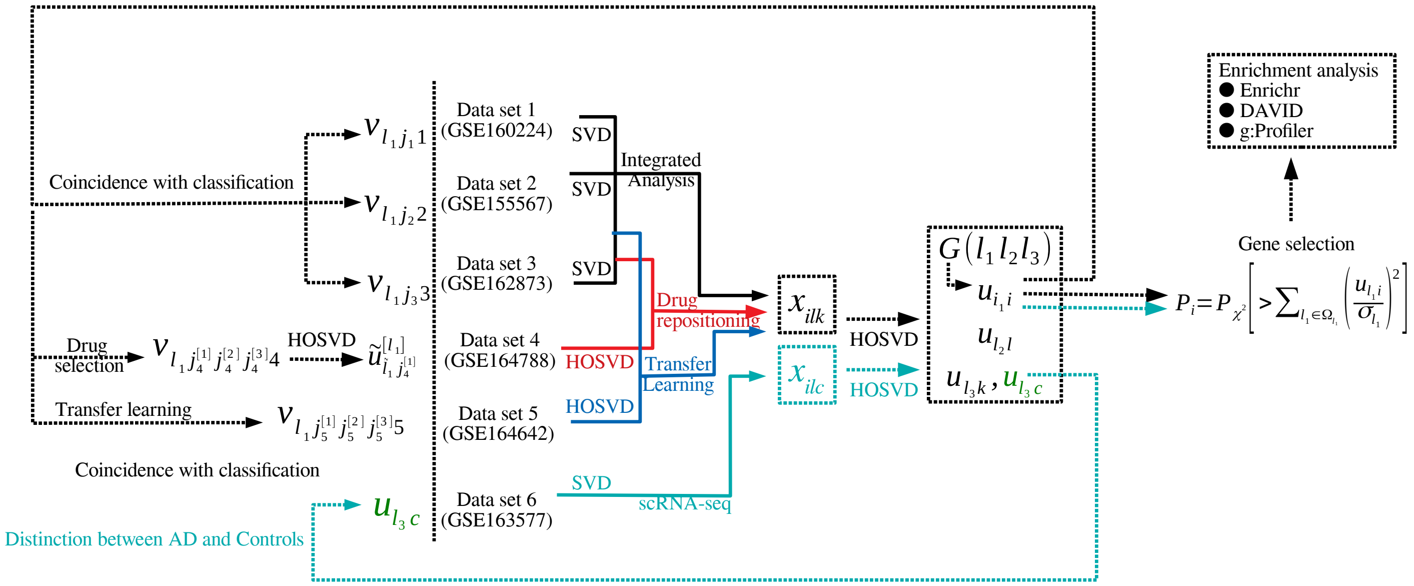

Figure S1: Schematic figure of the present analyses. At first, datasets 1, 2, and 3 were formatted as a tensor (black solid line); then, they were formatted as a tensor together with dataset 4 for drug repositioning (red solid line), and were also formatted as a tensor together with dataset 5 (blue solid line). Dataset 6 was separately formatted as a tensor (green solid line). HOSVD was applied to the generated tensors ($x_{i\ell k}$ or $x_{i\ell c}$). $u_{\ell_1 i}$s were used to evaluate coincidence with classification (left part of the figure) for datasets 1, 2, 3, 4, and 5 after being converted to $v_{\ell_1 j_k k}, 1 \leq k \leq 3$ or $v_{\ell_1 j_k^{[1]} \cdots j_k^{[S]} k}, k = 4, 5$. $u_{\ell_3 c}$ was used for the evaluation of the distinction between AD and controls for dataset 6 (green broken line, left part). $u_{\ell_1 i}$ was also selected in consideration of $\sum_{\ell, \ell_3} G(\ell_1 \ell \ell_3)^2$. The selected $u_{\ell_1 i}$s were used to attribute P-values to genes and genes were selected using P-values. Enrichment analyses were performed using selected genes by Enricher, DAVID, or g:Profiler (right part of the figure).

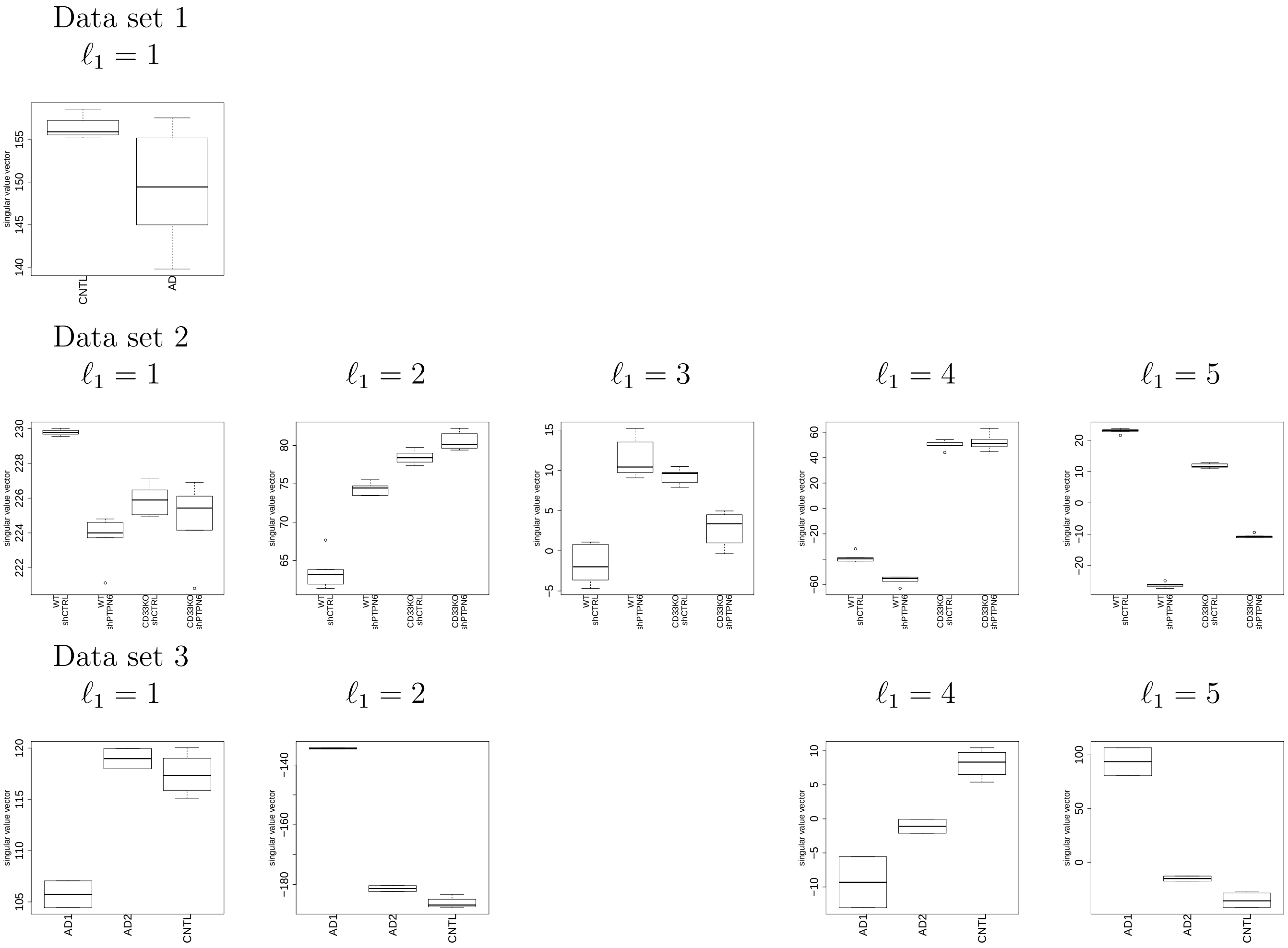

Figure S2: Boxplots of $v_{\ell_1 j_k k}$ for integrated analysis of datasets 1, 2 and 3. Top: $k = 1$, middle $k = 2$, bottom $k = 3$.

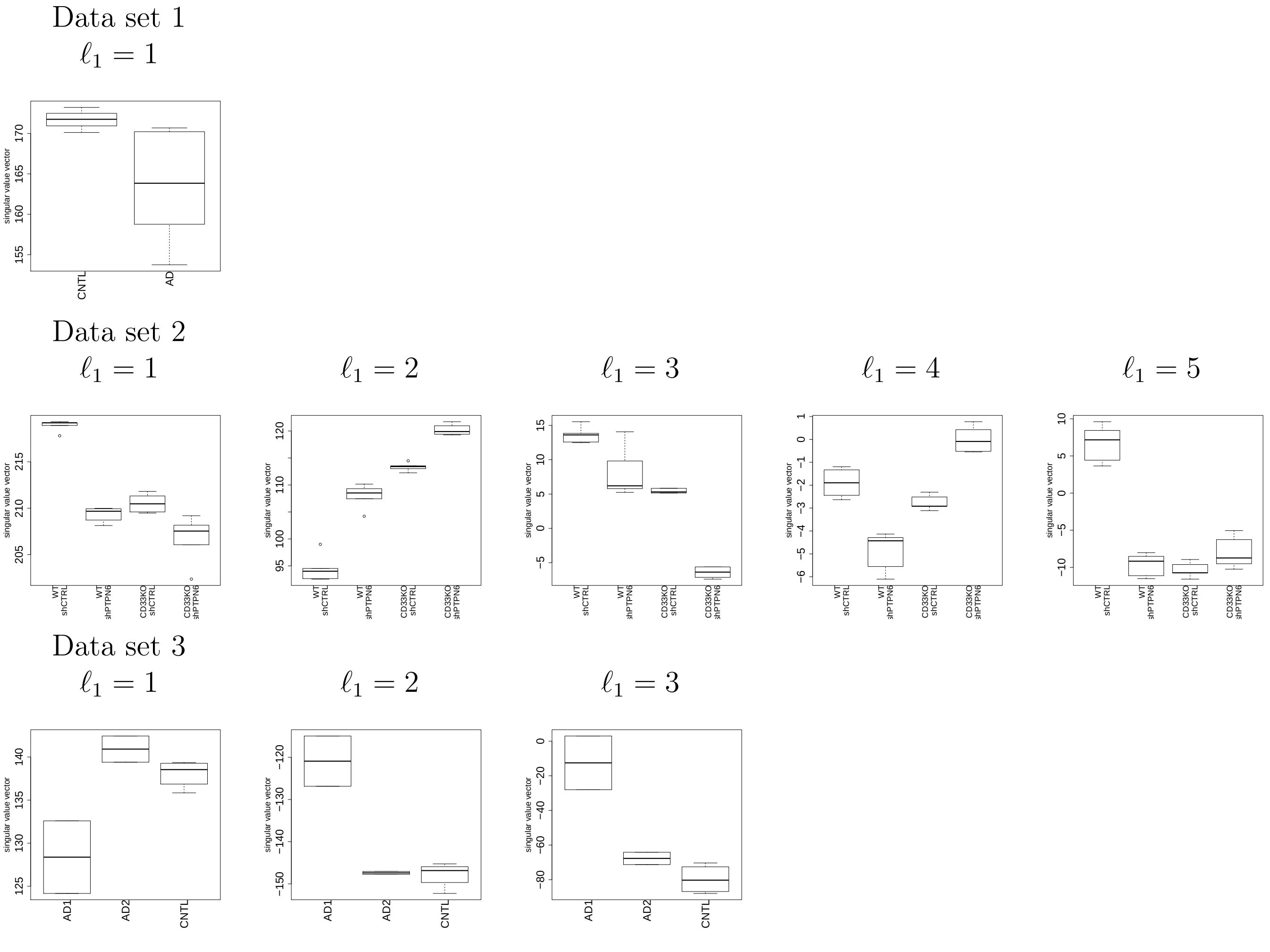

Figure S3: Boxplots of $v_{\ell_1 j_k k}$ for drug repositioning using the tensor obtained with data sets 1, 2 and 3. Top: $k = 1$, middle $k = 2$, bottom $k = 3$.

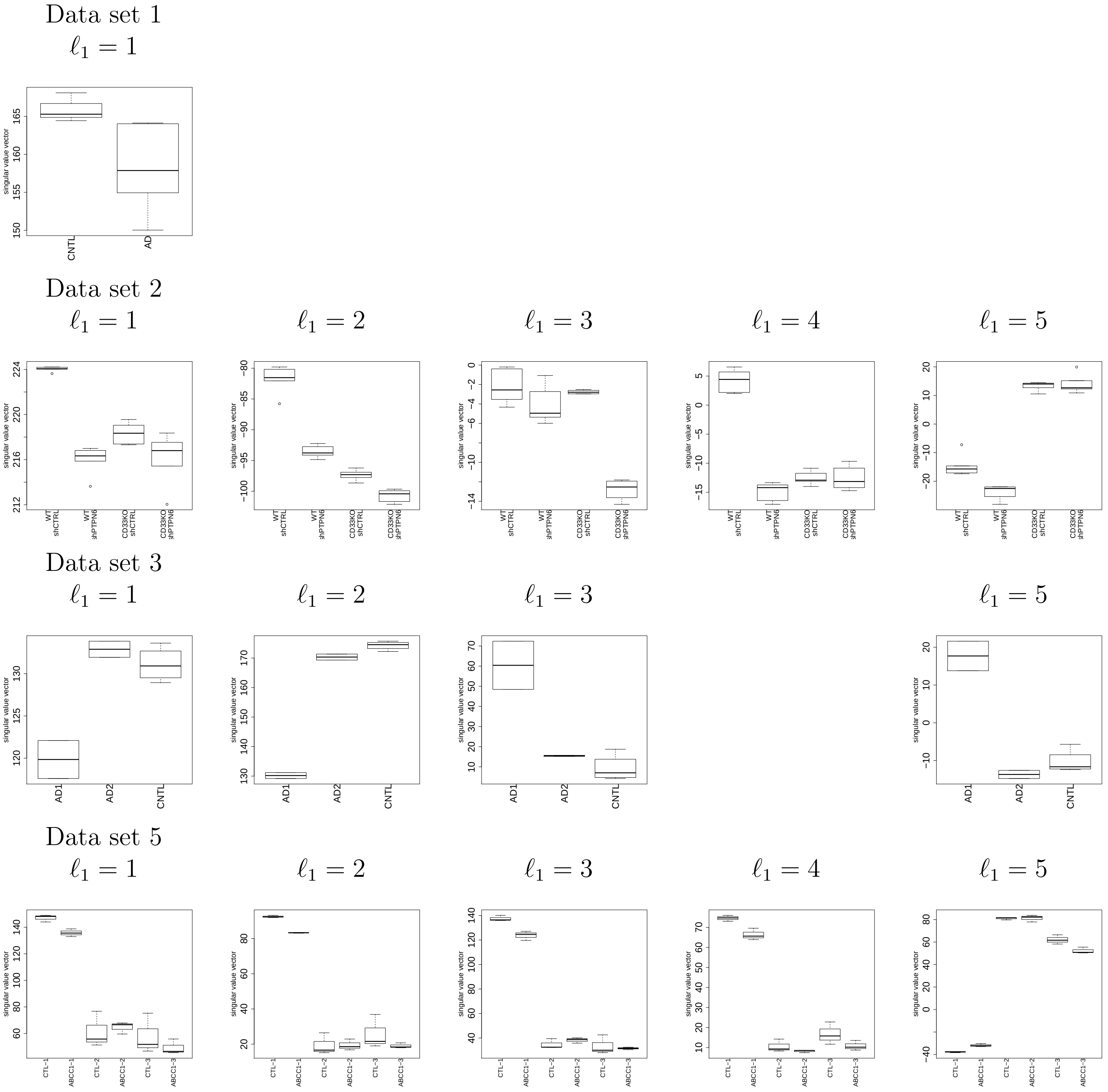

Figure S4: Boxplots of $v_{\ell_1 j_k k}$ (Top row: $k = 1$, second top row $k = 2$, third top row $k = 3$) and $v_{\ell_1 j_5^{[1]} j_5^{[2]} j_5^{[3]} 5}$ (Bottom row) for transfer learning using the tensor obtained with data sets 1, 2and 3

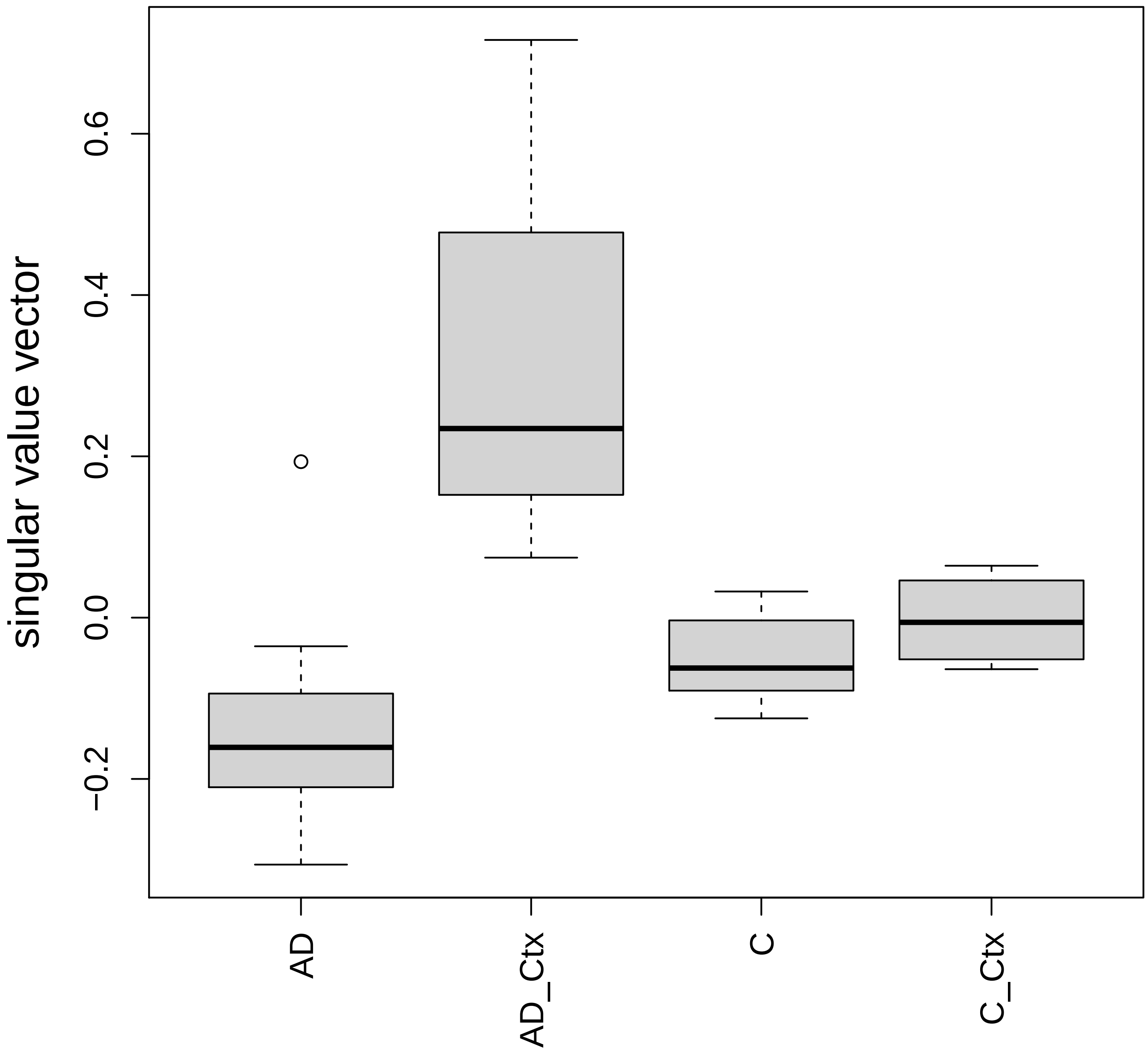

Figure S5: Boxplots of singular value vector, u_{6c}, computed by applying HOSVD to $x_{i\ell c}$ for scRNA-seq analysis. "AD" and "C" correspond to the hippocampus of patients and healthy controls, respectively, "AD_Ctx" and "C_Ctx" represent the cortex of patients and healthy controls, respectively.

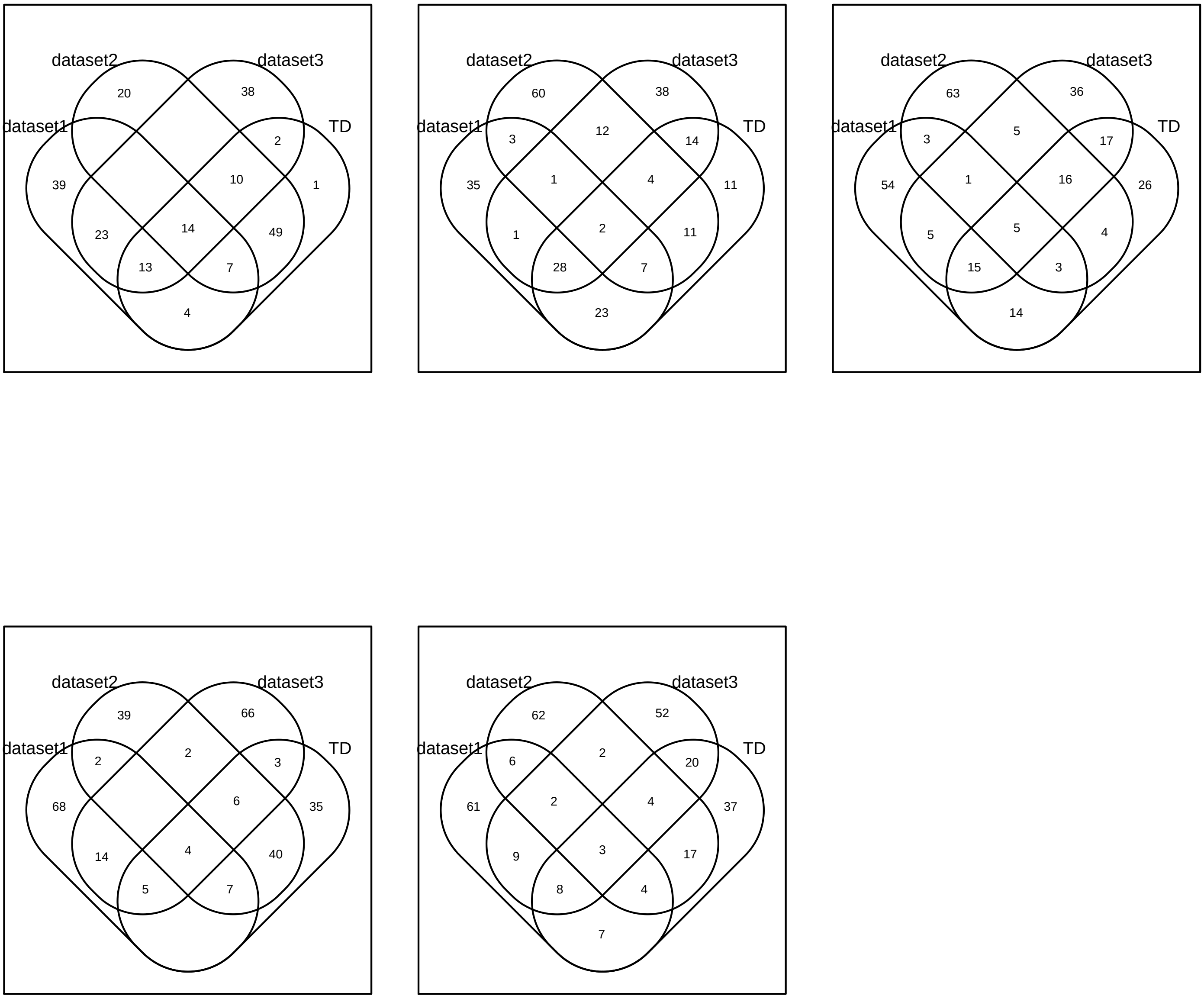

Figure S6: Venn diagram of top ranked 100 genes with larger absolute values of PC scores or singular value vectors. Upper row: from left to right, the first, the second and the third components. Bottom row: from left to right, the fourth and the fifth components
