# Supplementary material for: A tensor decomposition-based integrated analysis applicable to multiple gene expression profiles without sample matching
Source: Sci Rep. 2022 Dec 8;12:21242. doi: 10.1038/s41598-022-25524-4 (PMC9732005; doi:10.1038/s41598-022-25524-4)
Supplement: Supplementary file 9 — Supplementary Information 9. [file 41598_2022_25524_MOESM9_ESM.pdf]

Table S1: Coincidence between $v_{\ell_1 j_k k}$ (for datasets 1, 2 and 3) or $v_{\ell_1 j_5^{[1]} j_5^{[2]} j_5^{[3]} 5}$ (for dataset 5) and classification. Row P-values computed with t test for two classes (data set 1) or categorical regression for more classes (data set 2, 3 and 5).

Integration of data set 1, 2 and 3					
ℓ_1	1	2	3	4	5
Data set 1	4.86×10^{-2}	—	—	—	—
Data set 2	1.94×10^{-6}	7.81×10^{-14}	4.15×10^{-9}	$< 2.2 \times 10^{-16}$	$< 2.2 \times 10^{-16}$
Data set 3	1.55×10^{-3}	8.23×10^{-7}	—	2.74×10^{-3}	7.77×10^{-5}
Drug repositioning					
ℓ_1	1	2	3	4	5
Data set 1	2.82×10^{-2}	—	—	—	—
Data set 2	4.04×10^{-11}	3.95×10^{-15}	2.05×10^{-12}	3.05×10^{-10}	1.62×10^{-12}
Data set 3	2.02×10^{-2}	2.09×10^{-2}	3.53×10^{-3}	—	—
Transfer learning					
ℓ_1	1	2	3	4	5
Data set 1	1.77×10^{-2}	—	—	—	—
Data set 2	1.00×10^{-8}	9.07×10^{-15}	1.15×10^{-10}	1.05×10^{-13}	2.33×10^{-15}
Data set 3	3.31×10^{-3}	9.14×10^{-7}	3.35×10^{-3}	—	4.57×10^{-4}
Data set 5	1.87×10^{-8}	2.05×10^{-10}	4.24×10^{-13}	4.69×10^{-12}	3.17×10^{-16}

References

[1] Steve Rodriguez et al. Machine learning identifies candidates for drug repurposing in alzheimer's disease. *Nature Communications*, 12(1), February 2021.

Table S2: Brain disease related terms within top ranked ten terms in various enrichment analyses

Term	(A) 565 genes rk	(A) adjusted P-values	(B) 544 genes rk	(B) adjusted P-values	(C) 660 genes rk	(C) adjusted P-values	(D) 147 genes rk	(D) adjusted P-values
KEGG 2021 Human, Enrichr								
Parkinson disease	3	9.77×10^{-16}	3	3.49×10^{-19}	3	3.72×10^{-15}	3	1.76×10^{-11}
Pathways of neurodegeneration	4	7.86×10^{-13}	4	1.76×10^{-16}	4	1.39×10^{-11}	8	5.76×10^{-9}
Amyotrophic lateral sclerosis	5	1.53×10^{-12}	6	3.75×10^{-14}	5	4.84×10^{-11}	6	4.97×10^{-9}
Alzheimer disease	7	3.62×10^{-11}	5	1.40×10^{-14}	6	2.58×10^{-10}	10	2.08×10^{-7}
Huntington disease	8	4.75×10^{-9}	8	2.41×10^{-10}	9	2.17×10^{-6}	—	—
Prion disease	9	4.77×10^{-9}	7	1.14×10^{-11}	8	4.51×10^{-8}	—	—
Jensen Diseases, Enrichr								
Frontotemporal dementia	2	2.36×10^{-4}	9	4.14×10^{-3}	6	3.41×10^{-3}	—	—
Dementia	3	2.36×10^{-4}	—	—	—	—	—	—
Neurodegenerative disease	8	1.32×10^{-3}	5	1.27×10^{-3}	2	4.07×10^{-4}	8	3.04×10^{-2}
Periventricular nodular heterotopia	9	1.32×10^{-3}	6	1.27×10^{-3}	10	3.42×10^{-3}	—	—
Cerebrovascular disease	—	—	7	2.65×10^{-3}	9	3.42×10^{-3}	—	—
Pick's disease	—	—	—	—	7	3.41×10^{-3}	—	—
GAD_DISEASE_CLASS, DAVID								
PSYCH	1	2.03×10^{-9}	1	5.59×10^{-6}	1	7.14×10^{-9}	—	—
NEUROLOGICAL	3	6.77×10^{-5}	4	6.52×10^{-4}	2	3.04×10^{-7}	9	7.95×10^{-4}
AGING	—	—	—	—	3	2.18×10^{-6}	—	—
GAD_DISEASE, DAVID								
cognitive ability	2	2.94×10^{-13}	1	3.74×10^{-14}	2	3.00×10^{-12}	4	6.02×10^{-14}
Alzheimer Disease |Ataxia |Diabetes Mellitus, Type 2 |Disease |Obesity |Parkinson Disease	4	5.86×10^{-11}	3	1.63×10^{-11}	4	2.50×10^{-10}	3	3.01×10^{-14}
Alzheimer's Disease	5	1.06×10^{-9}	5	5.66×10^{-8}	5	8.44×10^{-8}	7	9.82×10^{-9}
Parkinson's Disease	6	1.14×10^{-7}	10	1.28×10^{-6}	9	3.74×10^{-6}	10	1.07×10^{-7}
Schizophrenia	8	4.35×10^{-7}	—	—	6	1.46×10^{-7}	—	—
KEGG, g:Profiler								
Parkinson disease	3	4.25×10^{-13}	3	1.06×10^{-14}	3	4.52×10^{-12}	3	1.59×10^{-7}
Amyotrophic lateral sclerosis	4	4.57×10^{-9}	6	2.54×10^{-9}	5	1.19×10^{-7}	7	6.78×10^{-5}
Pathways of neurodegeneration - multiple diseases	5	5.53×10^{-9}	4	5.41×10^{-11}	4	1.19×10^{-7}	9	2.58×10^{-4}
Alzheimer disease	6	2.77×10^{-8}	5	3.25×10^{-10}	6	3.07×10^{-7}	—	—
Prion disease	8	9.64×10^{-7}	7	5.38×10^{-8}	7	1.38×10^{-5}	10	6.29×10^{-4}
Huntington disease	9	1.57×10^{-6}	9	1.23×10^{-6}	9	5.84×10^{-4}	—	—

Terms are listed only when they are ranked upto top ranked ten terms. rk: rank, (A) Integrated analysis of data set 1, 2 and 3. (B) Drug repositioning (C) Transfer learning (D) Simple concatenation to which SVD was applied

Table S3: Top ranked five drugs. Those asterisked are identified as effective drugs in the original study [1] (We do not intend to mean that this table is adapted from Ref [1] but simply intend to mean that those asterisked are included in Ref [1]).

ℓ_1	Drugs
1	"ruxolitinib"*, "baricitinib* + lipofectamine", "a443654"*, "ldn-193189", "xmd16-144"
2	"ruxolitinib"*, "a443654"*, "baricitinib* + lipofectamine", "ldn-193189", "baricitinib* + dsRNA"
3	"ruxolitinib"*, "a443654"*, "xmd16-144", "baricitinib* + dsRNA", "ldn-193189"
4	"a443654"*, "baricitinib* + lipofectamine", "ldn-193189", "gsk1059615"*, "vorinostat"*
5	"a443654"*, "baricitinib* + lipofectamine", "gsk1059615"*, "bosutinib","xmd16-144"

Table S4: Enricment analysis for scRNA-seq

Human Gene Atlas Terms	adjusted P-values				
PrefrontalCortex	2.18×10^{-17}				
Amygdala	1.10×10^{-10}				
OccipitalLobe	1.12×10^{-5}				
pineal night	3.21×10^{-5}				
Fetalbrain	7.69×10^{-5}				
pineal day	4.27×10^{-4}				
SuperiorCervicalGanglion	7.48×10^{-4}				
Cerebellum	2.91×10^{-3}				
Wholebrain	8.13×10^{-3}				
CerebellumPeduncles	8.13×10^{-3}				
Disease	disease ID	species	GEO ID	sample	adj. P-values
Disease Perturbations from GEO down					
schizophrenia	DOID-5419		GSE25673	892	4.39×10^{-7}
Breast Cancer	C0006142		GSE1378	52	4.39×10^{-7}
Alzheimer's disease	DOID-10652	human	GSE5389	592	1.06×10^{-6}
Bipolar Disorder	C0005586		GSE5389	302	2.29×10^{-6}
schizophrenia	DOID-5419		GSE25673	891	2.81×10^{-6}
Alzheimer's disease	DOID-10652		GSE36980	520	3.46×10^{-6}
glaucoma associated with systemic syndromes	DOID-1686	mouse	GSE26299	488	2.06×10^{-18}
prostate cancer	DOID-10283	human	GSE26910	603	1.50×10^{-4}
astrocytoma	DOID-3069		GSE15824	860	2.01×10^{-4}
Huntington's disease	DOID-12858	mouse	GSE3621	704	2.22×10^{-4}
Disease Perturbations from GEO up					
Spinal Muscular Atrophy	C0026847	mouse	GSE10599	235	6.88×10^{-32}
multiple sclerosis	DOID-2377		GSE38010	737	6.72×10^{-7}
schizophrenia	DOID-5419		GSE25673	892	2.46×10^{-6}
multiple sclerosis	DOID-237		GSE38010	738	5.34×10^{-4}
schizophrenia	DOID-5419		GSE25673	891	7.63×10^{-4}
Schizophrenia	C0036341	human	GSE12649	261	1.72×10^{-4}
schizophrenia	DOID-5419		GSE49036	542	5.31×10^{-3}
Lewy body dementia	DOID-12217		GSE19587	1067	5.84×10^{-3}
polycystic ovary syndrome	DOID-11612		GSE48301	569	9.43×10^{-3}
Parkinson's disease	DOID-14330		GSE7621	941	1.32×10^{-3}

Table S5: $\sum_{\ell_2=1}^{3} G(\ell_1 \ell_2 \ell_3)^2$ for integrated analysis of data sets 1, 2 and 3. Although there are no specific criterion on how we can select ℓ_1 with this kind of table. In this case, since we cannot see any clear cut-off, we selected $1 \leq \ell_1 \leq 5$ Ω_{ℓ_1} tentatively. As can be seen in the paper, this selection is quite successful.

	ℓ_3			
ℓ_1	1	2	3	total
1	1.488130e+06	646.17367	224.8258	1489000.6891
2	5.753591e+02	614309.00484	661.5103	615545.8742
3	3.790016e+01	1344.92812	153264.1179	154646.9461
4	4.954126e+04	7644.18364	583.6019	57769.0421
5	8.795285e+03	17833.89058	6931.5236	33560.6996
6	1.520925e+04	3662.30058	4136.2153	23007.7615
7	3.389094e+02	1710.66077	3195.6861	5245.2563
8	1.429139e+02	6421.62945	4824.0816	11388.6249
9	4.446115e+02	1078.78354	2843.5224	4366.9175
10	8.540596e+00	22.99503	170.3017	201.8373

Table S6: $\sum_{\ell_2=1}^{4} G(\ell_1 \ell_2 \ell_3)^2$ for drug repositioning. Although there are no specific criterion on how we can select ℓ_1 with this kind of table. In this case, since we cannot see any clear cut-off, we selected $1 \leq \ell_1 \leq 5$ Ω_{ℓ_1} tentatively. As can be seen in the paper, this selection is quite successful.

	ℓ_3				
ℓ_1	1	2	3	4	total
1	1425610.4292	165491.7612	43058.646	51.77587	1634212.61227
2	51980.1280	322495.9243	376705.447	399.96152	751581.46082
3	22007.6757	151940.2455	140146.070	10723.91977	324817.91097
4	872.2898	121190.6967	27275.866	16034.31172	165373.16422
5	1445.7039	14865.0211	5491.229	127412.04217	149213.99617
6	43672.7337	4682.6374	7549.453	266.68380	56171.5079
7	5389.0112	3763.8854	12324.315	9032.87475	30510.08635
8	16304.1055	371.6352	3043.586	2234.39228	21953.71898
9	643.7475	13014.7429	4589.932	691.98229	18940.40469
10	1857.9075	624.5744	3193.985	6491.29191	12167.75881

Table S7: $\sum_{\ell_2 \in (1,3,4,5)} G(\ell_1 \ell_2 \ell_3)^2$ for transfer learning. Although there are no specific criterion on how we can select ℓ_1 with this kind of table. In this case, since we cannot see any clear cut-off, we selected $1 \leq \ell_1 \leq 5$ for Ω_{ℓ_1} tentatively. As can be seen in the paper, this selection is quite successful.

	ℓ_3				
ℓ_1	1	2	3	4	total
1	1555807.5603	2309.739	302.7296	98.90429	1558518.93319
2	4650.9970	618773.494	2449.1731	284.00856	626157.67266
3	3196.0969	2566.946	85538.0838	44028.71348	135329.84018
4	2050.7257	2369.817	8055.1904	103016.83892	115492.57202
5	4518.1605	7286.896	32743.2252	1490.78870	46039.0704
6	41238.9301	8194.855	2509.5804	915.94513	52859.31063
7	7287.5214	14243.817	2363.1287	6637.50175	30531.96885
8	13991.7789	3789.199	412.3858	4468.64273	22662.00643
9	1926.8077	3122.396	682.5117	6986.74539	12718.46079
10	296.7091	5736.129	275.3507	4893.82195	11202.01075

Table S8: $\sum_{\ell_2=1}^{10} G(\ell_1 \ell_2 6)^2$ for scRNA-seq. Since it takes the largest value for $\ell_1 = 6$, $\ell_1 = 6$ is selected as Ω_{ℓ_1}.

ℓ_1	
1	4361679
2	2110675
3	2932900
4	10004661
5	12247540
6	29645142
7	15351807
8	6629440
9	1832992
10	7382396

Table S9: Coincidence between latent variables and classification

CMF				
Latent variables	1st	2nd	3rd	4th
Data set 1	—	—	—	—
Data set 2	—	—	—	—
Data set 3	—	—	—	—
**GFA**				
Latent variables	1st	2nd	3rd	4th
Data set 1	—	—	—	—
Data set 2	1.94×10^{-6}	7.81×10^{-14}	4.15×10^{-9}	$< 2.2 \times 10^{-16}$
Data set 3	—	3.40×10^{-4}	1.26×10^{-2}	3.41×10^{-4}
**Simple concatenation**				
Latent variables	1st	2nd	3rd	4th
Data set 1	4.86×10^{-2}	—	—	—
Data set 2	6.88×10^{-5}	$< 2.2 \times 10^{-16}$	$< 2.2 \times 10^{-16}$	$< 2.2 \times 10^{-16}$
Data set 3	1.55×10^{-3}	8.23×10^{-7}	—	2.74×10^{-3}

Table S10: Classification performance for Integration of data set 1, 2 and 3 in Table S1

Dataset 1 $\ell_1 = 1$

		True	
		CNTL	AD
Predict	CNTL	3	2
	AD	0	4

Dataset 2 $\ell_1 = 1$

			True			
			WT		CD33KO	
		sh	CNTL	PTPN6	CNTL	PTPN6
predict	WT	CNTL	6	0	0	0
		PTPN6	0	4	0	2
	CD33KO	CNTL	0	0	3	4
		PTPN6	0	2	2	0

$\ell_1 = 2$

			True			
			WT		CD33KO	
		sh	CNTL	PTPN6	CNTL	PTPN6
predict	WT	CNTL	6	0	0	0
		PTPN6	0	6	0	0
	CD33KO	CNTL	0	0	4	1
		PTPN6	0	0	1	5

$\ell_1 = 3$

			True			
			WT		CD33KO	
		sh	CNTL	PTPN6	CNTL	PTPN6
predict	WT	CNTL	4	0	0	1
		PTPN6	0	3	1	0
	CD33KO	CNTL	0	3	4	0
		PTPN6	2	0	0	5

$\ell_1 = 4$

			True			
			WT		CD33KO	
		sh	CNTL	PTPN6	CNTL	PTPN6
predict	WT	CNTL	6	0	0	0
		PTPN6	0	6	0	0
	CD33KO	CNTL	0	0	3	3
		PTPN6	0	0	2	3

$\ell_1 = 5$

			True			
			WT		CD33KO	
		sh	CNTL	PTPN6	CNTL	PTPN6
predict	WT	CNTL	6	0	0	0
		PTPN6	0	6	0	0
	CD33KO	CNTL	0	0	5	0
		PTPN6	0	0	0	6

Table S10: (Continued) Classification performance for Integration of data set 1, 2 and 3 in Table S1

Dataset 3	$\ell_1 = 1$			
			True	
		AD1	AD2	CNTL
predict	AD1	2	0	0
	AD2	0	1	1
	CNTL	0	1	3
	$\ell_1 = 2$			
			True	
		AD1	AD2	CNTL
predict	AD1	2	0	0
	AD2	0	2	1
	CNTL	0	0	3
	$\ell_1 = 4$			
			True	
		AD1	AD2	CNTL
predict	AD1	1	0	0
	AD2	1	2	0
	CNTL	0	0	4
	$\ell_1 = 5$			
			True	
		AD1	AD2	CNTL
predict	AD1	2	0	0
	AD2	0	2	0
	CNTL	0	0	4

Table S11: Pearson's correlation coefficients between PC score attributed to genes and $u_{\ell_1 i}$ for Integration of datasets 1,2 and 3 in Table S1

ℓ_1		1			2		
		dataset			dataset		
		2	3	$u_{\ell_1 i}$	2	3	$u_{\ell_1 i}$
	1	3.77×10^{-1}	-6.72×10^{-1}	6.30×10^{-1}	-1.86×10^{-2}	-1.47×10^{-1}	-3.18×10^{-1}
dataset	2		-2.50×10^{-1}	9.50×10^{-1}		3.26×10^{-2}	-9.06×10^{-2}
	3			-4.98×10^{-1}			1.57×10^{-1}
ℓ_1		3			4		
		dataset			dataset		
		2	3	$u_{\ell_1 i}$	2	3	$u_{\ell_1 i}$
	1	-4.72×10^{-3}	-1.45×10^{-1}	-3.83×10^{-1}	-1.08×10^{-2}	-7.85×10^{-2}	-1.03×10^{-1}
dataset	2		7.63×10^{-2}	-1.09×10^{-1}		1.20×10^{-1}	-7.69×10^{-3}
	3			1.94×10^{-1}			8.34×10^{-2}
ℓ_1		5					
		dataset					
		2	3	$u_{\ell_1 i}$			
	1	4.76×10^{-2}	1.20×10^{-1}	-1.29×10^{-1}			
dataset	2		-1.26×10^{-2}	2.14×10^{-2}			
	3			-3.66×10^{-2}			
